# Prescription of oral antidiabetic drugs in Tyrol – Data from the Tyrol diabetes registry 2012–2015

**DOI:** 10.1007/s00508-016-1135-1

**Published:** 2016-12-01

**Authors:** Lukas Lunger, Andreas Melmer, Willi Oberaigner, Marco Leo, Martin Juchum, Karin Pölzl, Johannes Gänzer, Martha Innerebner, Egon Eisendle, Gertrud Beck, Hermann Kathrein, Bernhard Heindl, Hans Robert Schönherr, Monika Lechleitner, Herbert Tilg, Christoph Ebenbichler

**Affiliations:** 1 Departement, Medical University of Innsbruck, Innsbruck, Austria; 2 Departement for Clinical Epidemiology of the Tiroler Landeskliniken, Tirol Kliniken, Innsbruck, Austria; 3 PKA, Private Hospital, Münster, Austria; 4 Departement for Internal Medicine, Hospital Natters, Natters, Austria; 5 Departement for Internal Medicine, Hospital Hall in Tirol, Hall in Tirol, Austria; 6 Departement for Internal Medicine, Hospital Kufstein, Kufstein, Austria; 7 Departement for Internal Medicine, Hospital Lienz, Lienz, Austria; 8 Departement for Internal Medicine, Hospital Reutte, Reutte, Austria; 9 Departement for Internal Medicine, Hospital Schwaz, Schwaz, Austria; 10 Departement for Internal Medicine, Hospital St. Johann in Tirol, St. Johann in Tirol, Austria; 11 Departement for Internal Medicine, St. Vinzenz Hospital, Zams, Austria; 12 Departement for Internal Medicine and Geriatrics, Hospital Hochzirl, Zirl, Austria

**Keywords:** Diabetes mellitus, Hypoglycemic Agents, Registries, Metformin, Insulins

## Abstract

Diabetes mellitus affects 9% of the adult population worldwide and the economic burden of the disease is growing exponentially. In type 2 diabetes mellitus (T2DM), when life style interventions fail to achieve treatment targets, oral antidiabetic drugs are prescribed to improve glycemic control. Several new oral antidiabetics have been launched in the last few years, which enlarged the spectrum of available treatment options in T2DM. The present study aimed to examine T2DM treatment patterns in a cohort of 7769 patients recruited from the Diabetes Registry Tyrol (DRT) with at least one visit from 2012–2015. Secondly, the study aimed to evaluate the use of new oral antidiabetics compared to older oral antidiabetics (OAD). It was found that 43.4% of all patients were treated with OAD alone while 21.2% had oral antidiabetics combined with insulin. 19.9% of the study population were treated with insulin or insulin analogs only. 15.3% had no pharmacological treatment. Metformin was used most frequently (47.9% of the study population), followed by gliptines (27.2%). The most common treatment regimen in this population was the dual therapy of metformin and another OAD (17.2%), followed by metformin monotherapy (16.6%) and triple therapy of metformin and two additional OAD (11.0%).

## Introduction

Diabetes mellitus is one of the most prominent causes of premature illness and death worldwide. Type 2 diabetes mellitus (T2DM) is characterized by increased insulin resistance, depleted insulin secretion and subsequently elevated levels of blood glucose. The T2DM affects approximately up to 6.4 % of adults in the general population; however, an estimated 50 % of cases of T2DM may still remain undiagnosed at present [1]. In Austria, T2DM is widespread and affects nearly 9 % of the population, which accounts for 640,000 inhabitants [2]. Main treatment goals for T2DM are to achieve near normal levels of blood glucose as well as to avoid acute and long term complications, among which hypoglycemia and hyperglycemia, diabetic nephropathy, diabetic retinopathy, and diabetic neuropathy are the most common [2, 3].

Treatment strategies for T2DM comprise life style modification, patient-centred education programs, and pharmacological treatment options. The American Diabetes Association (ADA) and the European Association for the Study of Diabetes (EASD) consensus statements recommended glycated hemoglobin A1C (HbA1c) levels <7 % as being desirable for most non-pregnant adults, due to the reduction of microvascular complications [4]; however, higher target values of HbA1c may be advisable in the presence of complications, age or comorbidities [5].

According to national and international guideline recommendations, pharmacological treatment options are recommended when life style and education programs fail to achieve HbA1c levels <6.5 %. In the absence of certain contraindications, oral antidiabetic treatment with metformin should be initiated, due to its favorable risk profile for hypoglycemic events, its beneficial influence on body weight as well as due to financial considerations. Treatment can be escalated twice up to the use of three oral antidiabetic drugs (OAD) or be combined with subcutaneous insulin substitution. The OAD should be chosen according to efficacy, side effects, patient-derived risk factors, treatment targets and availability. Newer OAD, namely glucagon-like peptide-1 (GLP-1) agonists and sodium-glucose cotransporter 2 (SGLT-2) inhibitors have expanded the spectrum of available treatment options [3, 5–7]. Pharmacological treatment accounts for a greater part of health economic costs related with T2DM, besides treatment of complications, clinical examination and absence from work due to complications.

The aim of the present study was to characterize the current prescription patterns of antidiabetic drugs in the region of Tyrol between 2012 and 2015; therefore, data from the diabetes registry of Tyrol (DRT) were analyzed in order to identify current developments of the treatment of T2DM in a real life situation.

## Patients and methods

The DRT was initiated in 2005 and includes relevant data from approximately 17,000 patients with diabetes mellitus, 11,000 of which were diagnosed with T2DM. The present analysis covers data from 7769 T2DM patients, who attended at least one T2DM-related outpatient or inpatient visit between 1 January 2012 and 31 December 2015. The DRT records demographic, anamnestic and clinical data of incident and prevalent T2DM patients. Relevant data comprise age, sex, body mass index (BMI), HbA1c, diabetes duration, diabetic long-term complications (e.g. diabetic nephropathy, neuropathy, retinopathy and macrovascular complications). Pharmacological treatment has been recorded since 2012, including substance class, start and end date of prescription. The date of diagnosis of T2DM was known in 5827 patients (approximately 75%). For each patient, the most recent examination was used for analysis between the year 2012 and 2015.

This study aimed to summarize antidiabetic medication prescription in T2DM patients who participated in the DRT between the years 2012–2015. As the current investigation did not aim to correlate or compare any prescription patterns between participants, no statistical tests were performed. Current prescription patterns were illustrated using STATA statistical analysis software, version 13 (StataCorp LP, College Station, TX).

## Results

Table 1 shows the demographic and clinical data of T2DM patients, Table 2 illustrates prescription patterns of OADs and insulin and Table 3 provides data on age-dependent prescription patterns of OADs and insulin. Table 4 shows data on insulin use in the DRT.

**Table 1:** Demographic and clinical data in the diabetes registry of Tyrol

Parameter	Patients (*n*)	Age (years, mean ± SD^a^)	Frequency (%)
Sex			
Male	3260	66.5 ± 12.9	42.0
Female	4509	64.2 ± 11.8	58.0
Total	7769	65.2 ± 12.3	100
Age groups (years)			
18–39	207	–	2.7
40–59	2229	–	28.7
60–79	4418	–	56.9
80–99	915	–	11.8
Diabetes duration (years)			
0–5	1824	–	29.8
5–10	1472	–	24.1
10–20	1999	–	32.7
20–99	822	–	13.4
Diabetes associated late complications			
Nephropathy	705	–	15.4
Neuropathy	472	–	10.3
Myocardial infarction	470	–	10.3
Apoplectic insults	287	–	6.3
Peripheral artery disease	258	–	5.7
Retinopathy	103	–	2.3

^a^Mean ± *SD* standard deviation

**Table 2:** Therapies in the treatment of type 2 diabetes mellitus in the diabetes registry of Tyrol

Class	Frequency *n* (%)
Oral	5018 (64.6)
Metformin	3722 (47.9)
Gliptins	2114 (27.2)
Metformin or gliptins	4387 (56.5)
Glitazones	289 (3.7)
Glucosidase inhibitors	33 (0.4)
SGLT-2 inhibitors	291 (3.7)
Sulfonylurea analogues	1046 (13.5)
GLP-1 analogues	83 (1.1)
Insulin	1335 (17.2)
Insulin analogues	2569 (33.1)
Insulin or analogues	3193 (41.1)
Bariatric surgery	32 (0.4)
Life style intervention only	1190 (15.3)
Total	7769 (100.0)

*SGLT-2* sodium-glucose cotransporter 2, *GLP-1* glucagon like peptide-1

**Table 3:** Therapies in the treatment of type 2 diabetes mellitus in the diabetes registry of Tyrol by age groups

Class	18–39 years, *n* (%)	40–59 years, *n* (%)	60–79 years, *n* (%)	80–99 years, *n* (%)
Oral	123 (59.4)	1592 (71.4)	2878 (65.1)	425 (46.4)
Metformin	114 (55.1)	1349 (60.5)	2077 (47.0)	182 (19.9)
Gliptins	40 (19.3)	653 (29.3)	1238 (28.0)	183 (20.0)
Metformin or gliptins	119 (57.5)	1467 (65.8)	2505 (56.7)	296 (32.3)
Glitazones	7 (3.4)	113 (5.1)	156 (3.5)	13 (1.4)
Glucosidase inhibitors	–	13 (0.6)	14 (0.3)	6 (0.7)
SGLT-2 inhibitors	12 (5.8)	128 (5.7)	150 (3.4)	1 (0.1)
Sulfonylurea analogues	14 (6.8)	237 (10.6)	627 (14.2)	168 (18.4)
GLP-1 analogues	1 (0.5)	37 (1.7)	43 (1.0)	2 (0.2)
Insulin	27 (13.0)	304 (13.6)	786 (17.8)	218 (23.8)
Insulin analogues	44 (21.3)	613 (27.5)	1552 (35.1)	360 (39.3)
Insulin or insulin analogues	52 (25.1)	754 (33.8)	1908 (43.2)	479 (52.3)
Bariatric surgery	7 (3.4)	15 (0.7)	10 (0.2)	–
Life style intervention only	54 (26.1)	342 (15.3)	643 (14.6)	151 (16.5)
Total	207 (100.0)	2229 (100.0)	4418 (100.0)	915 (100.0)

*SGLT‑2* sodium-glucose cotransporter 2, *GLP‑1* glucagon like peptide‑1

**Table 4:** Insulin use in the diabetes registry of Tyrol

Treatment type	Frequency *n* (%)
OAD without insulin	3370 (43.4)
OAD with insulin	1648 (21.2)
Insulin therapy only (classical and/or analogue)	1544 (19.9)
No therapy	1190 (15.3)
Total	7769 (100.0)

*OAD *

Of 7690 patients analyzed, 58% were female, 42% were male and the mean age was 65.2 ± 12.3 years. Of the patients 56.9% where in the age group 60–79 years, 28.7% were aged between 40–59 years, 11.8% were older than 80 years and 2.7% were aged below 39 years. The mean BMI was 30.1 ± 6.1 kg/m^2^ and mean HbA1c in the study population was 7.6% ± 1.44. The most common diabetes duration was 10–20 years (32.7%), followed by 0–5 years (29.8%) and 5–10 years (24.1%). Least frequent in the DRT were patients with a diabetes duration over 20 years (13.4%). Of the diabetes associated late complications, nephropathy was the most common (15.4%), followed by neuropathy (10.3%) and myocardial infarction (10.3%). Less common were apoplectic insults (6.3%), peripheral artery disease (5.7%) and retinopathy (2.3%). Of all the patients analyzed 85% received at least one type of T2DM-related treatment other than lifestyle education (pharmacological or surgical treatment). In 43.4% of all patients OAD alone were used, followed by OAD combined with insulin (21.2%), resulting in a total of 65% of patients who used at least one OAD. Single use of insulin (classical insulin and insulin analogues) was prescribed in 19.9% of all patients analyzed. Among OADs prescribed in the DRT, metformin was the most frequent (47.9%), followed by gliptines (27.2%), sulfonylurea analogues (13.5%), glitazones (3.7%), SGLT-2 inhibitors (3.7%), GLP-1 analogues (1.1%) and glucosidase inhibitors (0.4%). Metformin monotherapy was used in 16.6% of all patients. Dual therapy (metformin + another OAD) was prescribed in 17.2% of all patients. Among those, the combination with gliptines (9.9%) was most common, followed by insulin (or insulin analogues 5.3%), and sulfonylurea analogues (2.0%). Triple therapy (metformin + 2 other OADs and/or insulin) was used in 11% of all patients. Among those, metformin plus gliptines plus another OAD was used most frequently (6.5%).

Metformin was most frequently prescribed in patients <60 years of age (55.1% in patients between 18–39 years and 60.5% in patients between 40–59 years). Older patients (≥60 years) had less prescription of metformin (47% in patients between 60–79 years and 19.9% in patients ≥80 years). The second most commonly prescribed OADs were gliptines, which were used in 19.3% of patients between 18–39 years, in 29.3% of patients between 40–59 years, 28% of patients between 60–79 years and 20% in older patients (≥80 years). The third most common OAD group were sulfonylurea analogues, which were predominantly prescribed in older patients (≥80 years). Newer treatment options (SGLT-2-inhibitors and GLP-1-analogues) were prescribed more rarely and predominantly in patients between 18–39 years of age. Use of insulin was more frequent in elderly patients with a peak among patients ≥80 years (23.8%) and 15.3% of T2DM patients did not receive any treatment except for education and life style modification.

## Discussion

In the DRT, metformin, either alone or as part of a combination therapy, was prescribed most frequently, followed by gliptines. The most common dual therapy was metformin plus gliptines, followed by metformin plus insulin. Sulfonylureas were used more frequently in older patients. Insulin therapy was used in approximately half of all T2DM patients in the DRT, newer OADs were used less frequently and more in younger T2DM patients. The majority of T2DM patients in this cohort were treated with well-established OADs but this may be explained by favorable safety profiles and efficacy. On the other hand constraints in reimbursement are still significant for some of the newer OADs, which may impede the prescription in an outpatient setting, even though scientific and clinical knowledge encourage the prescription of newer OADs.

Treatment strategies for T2DM vary worldwide depending on the prescribed substances, reimbursement regulations and lifestyle education, factors that are likely driven by regional financial capacity and epidemiological profiles. These international differences are most impressively illustrated by data from recent epidemiological studies, which show that most T2DM patients are living in developing or transition countries [8, 9]. The increasing prevalence of T2DM as well as the development of new OADs emphasize the need for feasible and cost-efficient treatment strategies in the long-term. Treatment guidelines vary across different countries, making regular epidemiological meta-analyses a reasonable tool of quality assurance and adaption. Although reimbursement regulations in most European countries allow a relatively broad spectrum of available treatment options, this does not apply for most non-European countries, especially in terms of newer OADs. Patients may be confronted with high retention fees, insufficient supplies or restrictions in available treatment options.

The national diabetes report of the USA showed that approximately 57% of all patients in 2014 were treated with OADs alone, while approximately 15% of all patients were given a combination of insulin and OADs. Insulin monotherapy was established in 14% of the patients. Compared to data from the USA, patients registered in the DRT were prescribed OADs less often (44.3%), while the combination of OAD and insulin was more common (21.2%). The most commonly prescribed OAD in the USA is currently metformin (used in approximately 50% of diabetes patients) [10, 11]. Over the years, partially influenced by the development of newer medications, prescription patterns have significantly changed. Since an analysis by Brouwer et al. [12] it was found that metformin prescription rates decreased from 60-70% in 2000 to current world-wide estimated rates of 45–50% [8], which is in line with our results; however, metformin monotherapy was prescribed only in 17% in the DRT compared to 53% in the USA [7]. A possible explanation is frequent use of fixed dose combinations (including metformin and another OAD) in the DRT. Also, pharmacological second line treatments (e. g. gliptines) are both less expensive and are reimbursed to a considerable amount by the healthcare system. Moreover, metformin is contraindicated in the presence of certain conditions and comorbidities, such as nephropathy, which was manifest in 15.4% of DRT patients. Another reason might be that the use of metformin is associated with a considerable frequency of adverse effects (including gastrointestinal complaints) and was found to induce weight loss, which may be limit the use for elderly, lean patients. In our study population 27% of all patients had gliptine medication and 57% were treated with either metformin or gliptines, while gliptines were only used in 8–13% of patients in the USA. On the other hand, sulfonylureas were used more frequently in the USA (23–27% of all T2DM patients), alone or as part of a combination therapy. In the DRT, however, sulfonylureas were the third most common OADs (13%) and were more frequently prescribed in older patients. In principle, it might be expected that insulin use would increase in older patients due to the longer duration of disease; however, insulin treatment may impose a challenge to older patients and their family members or caretakers. Insulin is considered the most effective and flexible therapy for T2DM but the adverse event profile, especially the risk for severe hypoglycemia and the need for cognition and compliance, make insulin a challenging treatment option for both patients and prescribers. While initial insulin treatment patterns in T2DM are relatively easy for compliance, increasing demands of insulin often require more complex strategies, such as multiple daily injections, intensified self-assessment of blood glucose, carbohydrate:insulin ratios; therefore, sulfonylureas may be an attractive alternative to the more complex and time-consuming insulin regimen. Insulin use over the age groups increased by 27.2% compared to an increase of sulfonylurea use of 11.6%; however, in younger patients the lower prescription rates of sulfonylureas compared to gliptines may be explained by the unfavorable risk profile and side effects [10, 13, 14]. The prescription of newer therapies, however, was less frequent in DRT. After stratification of the cohort by age and subsequent analyses, it was shown that these treatments are more frequently given to younger patients, which is in line with international data [7, 10]. The less frequent prescription of newer OADs may be explained by their current prescription costs and higher bureaucratic effort. For instance, treatment costs for T2DM medications increased by 61% due to the implemented use of insulin glargine and gliptines in the USA.

The prevalence of diabetic late complications was generally lower compared to data from the USA. Among patients in the DRT, nephropathy was most common (15.4%), followed by neuropathy (10.3%) and myocardial infarction (10.3%). In the USA, nephropathy is reported to occur in approximately 40% of diabetes patients [15–17]. The prevalence of peripheral neuropathy is estimated to occur in 12–50% of diabetic patients [18]; however, sufficient epidemiological data from the USA are not available to date. Therefore, the ADA reported prevalence rates refer to European data [18]. In the DRT, myocardial infarction occurred in 10.3% and apoplectic insults in 6.3% of participants. The age-adjusted percentage of people aged 35 years or older with diabetes and with self-reported coronary heart disease (i. e. self-reported coronary heart disease, angina or heart attack) was more than two times that of self-reported stroke (21.9% vs. 9.1%) [19]. The true prevalence of peripheral artery disease (PAD) is difficult to estimate due to its coincidence with peripheral neuropathy, which may mitigate pain perception and the high percentage of asymptomatic patients. Amputation has been used as a measure for PAD; however, medical care and local indications for amputation vary widely. The current nationwide, age-adjusted amputation rate is prevalent in 3% of US inhabitants. Retinopathy was present in 2.3% of DRT participants. According to data from the governmental National Eye Institute, approximately 8.4% of diabetic patients in the USA were diagnosed with some degree of retinopathy [20].

In conclusion, we found that metformin is the most commonly prescribed OAD in the DRT, while newer OADs are prescribed less frequently and more in younger T2DM patients. Sulfonylureas are still a popular option, especially in older T2DM patients.
